# Supplementary material for: Methanogenesis and Salt Tolerance Genes of a Novel Halophilic Methanosarcinaceae Metagenome-Assembled Genome from a Former Solar Saltern
Source: Genes (Basel). 2021 Oct 13;12(10):1609. doi: 10.3390/genes12101609 (PMC8535929; doi:10.3390/genes12101609)
Supplement: Supplementary file 1 [file genes-12-01609-s001.zip › genes-1410471-sup.pdf]

Supplementary Material for:

Methanogenesis and salt tolerance genes of a novel halophilic Methanosarcinaceae metagenome-assembled genome from a former solar saltern

Clifton P. Bueno de Mesquita[1], Jinglie Zhou[1], Susanna Theroux[1,2], Susannah G. Tringe[1,3,*]
[1]Department of Energy Joint Genome Institute, Lawrence Berkeley National Laboratory, Berkeley, CA, USA
[2]Southern California Coastal Water Research Project, Costa Mesa, CA, USA
[3]Environmental Genomics and Systems Biology Division, Lawrence Berkeley National Laboratory, Berkeley, CA, USA
*Corresponding author sgtringe@lbl.gov

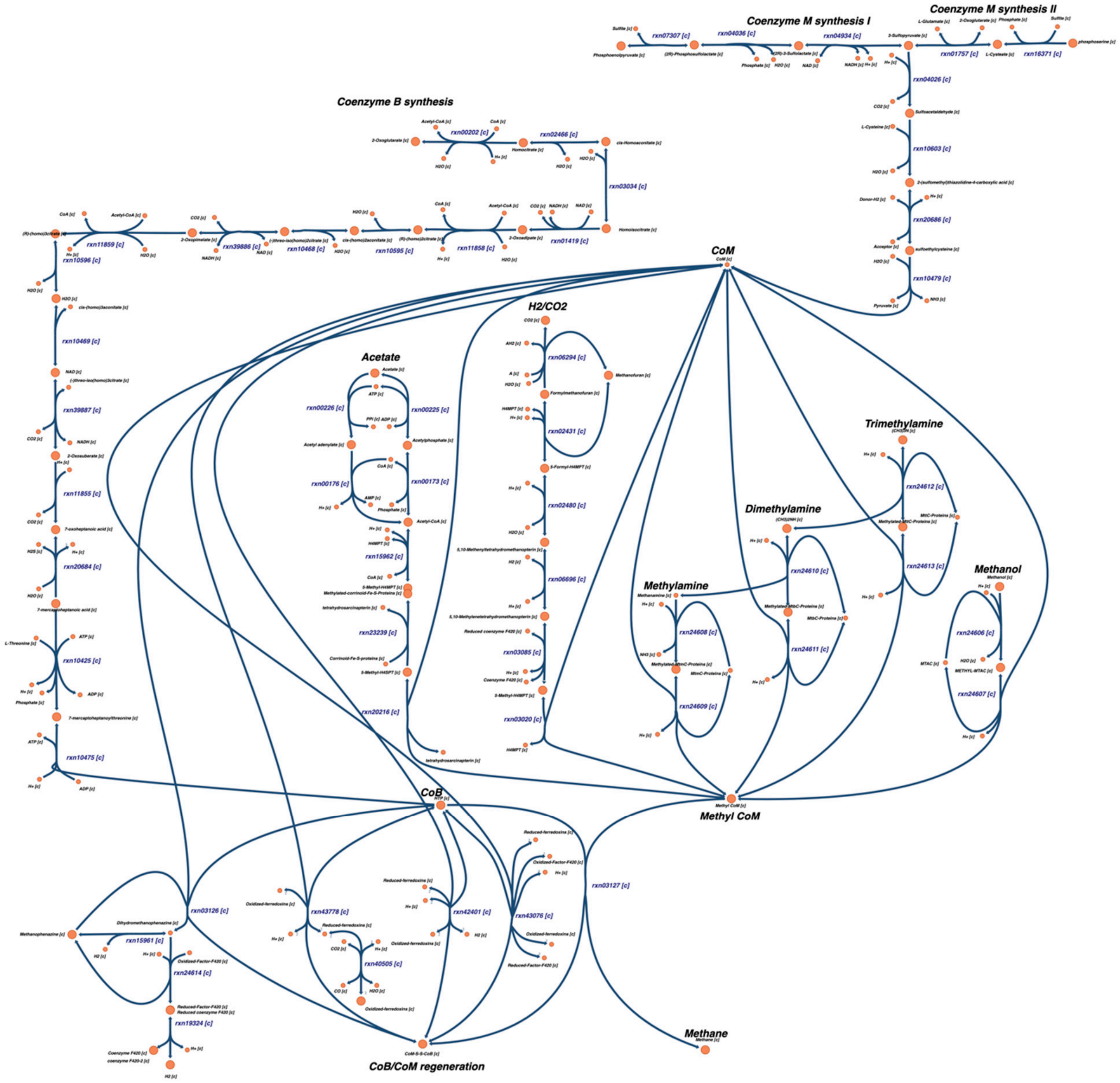

Figure S1. Map of several important pathways in methanogenesis including synthesis of coenzyme B (CoB) and coenzyme M (CoM), formation of methyl CoM from either acetate, hydrogen and carbon dioxide, methylamine, dimethylamine, trimethylamine or methanol, reduction of methyl CoM to methane, producing heterodisulfide as a byproduct, and CoB/CoM regeneration from heterodisulfide. Shown are the ModelSeed reaction IDs and compound names. The [c] next to the reactions and compounds denotes that it occurs in the cytoplasm. Not shown are pathways for methyl CoM formation from tetramethylammonium, dimethyl sulfide, glycine betaine, methanethiol, which are not annotated with KOs.

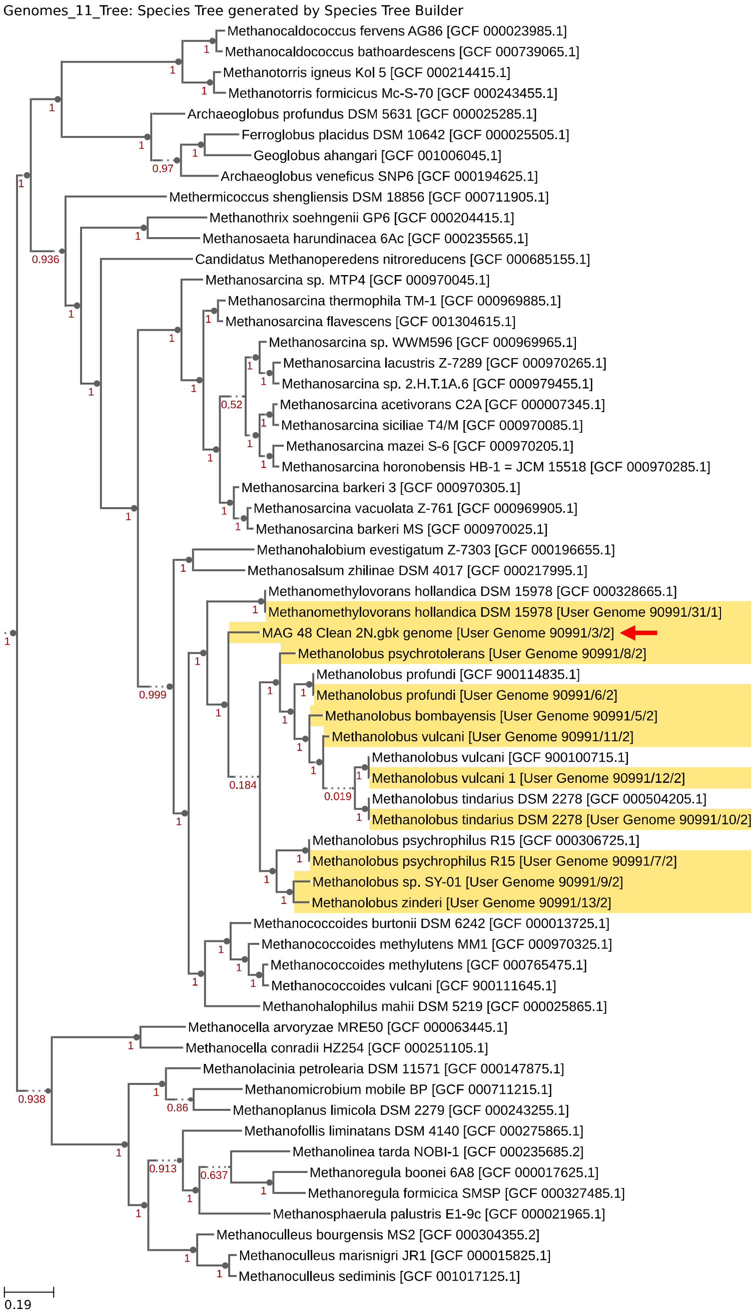

Figure S2. The 11 genomes analyzed in this study (highlighted) placed within a reference tree set to display 50 other genomes. The tree was built using the “Insert Set of Genomes into SpeciesTree” tool in Kbase, which uses 49 single copy COGs to build the tree. Note that “MAG 48 Clean 2N = *Methanosalis* sp. SBSPR1A, which is marked with a red arrow; *Methanolobus vulcani* = *Methanolobus vulcani* B1d; *Methanolobus vulcani* 1 = *Methanolobus vulcani* PL 12/M. The scale bar refers to substitutions/site and the red numbers refer to branch support values based on the Shimodaira-Hasegawa test.

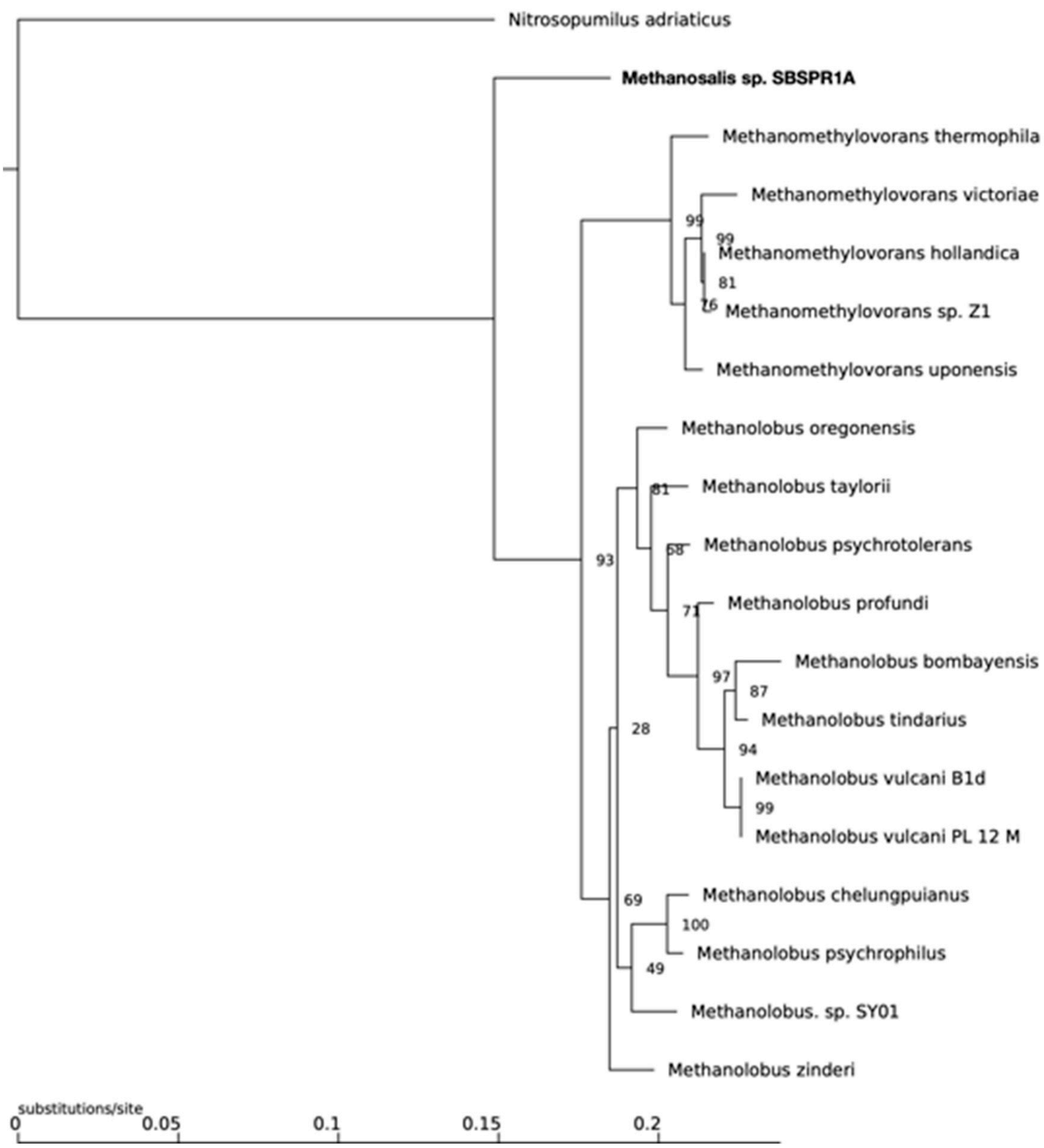

Figure S3. Phylogenetic tree of full length 16S rRNA genes extracted from the 11 complete genomes in this study plus seven additional taxa with available 16S rRNA genes on NCBI. Branch labels show the bootstrap support, calculated with 1000 bootstraps. The Methanosalis sp. SBSPR1A genome presented in this paper is highlighted in bold. The tree was built with the IQ-TREE online program (http://www.iqtree.org/).

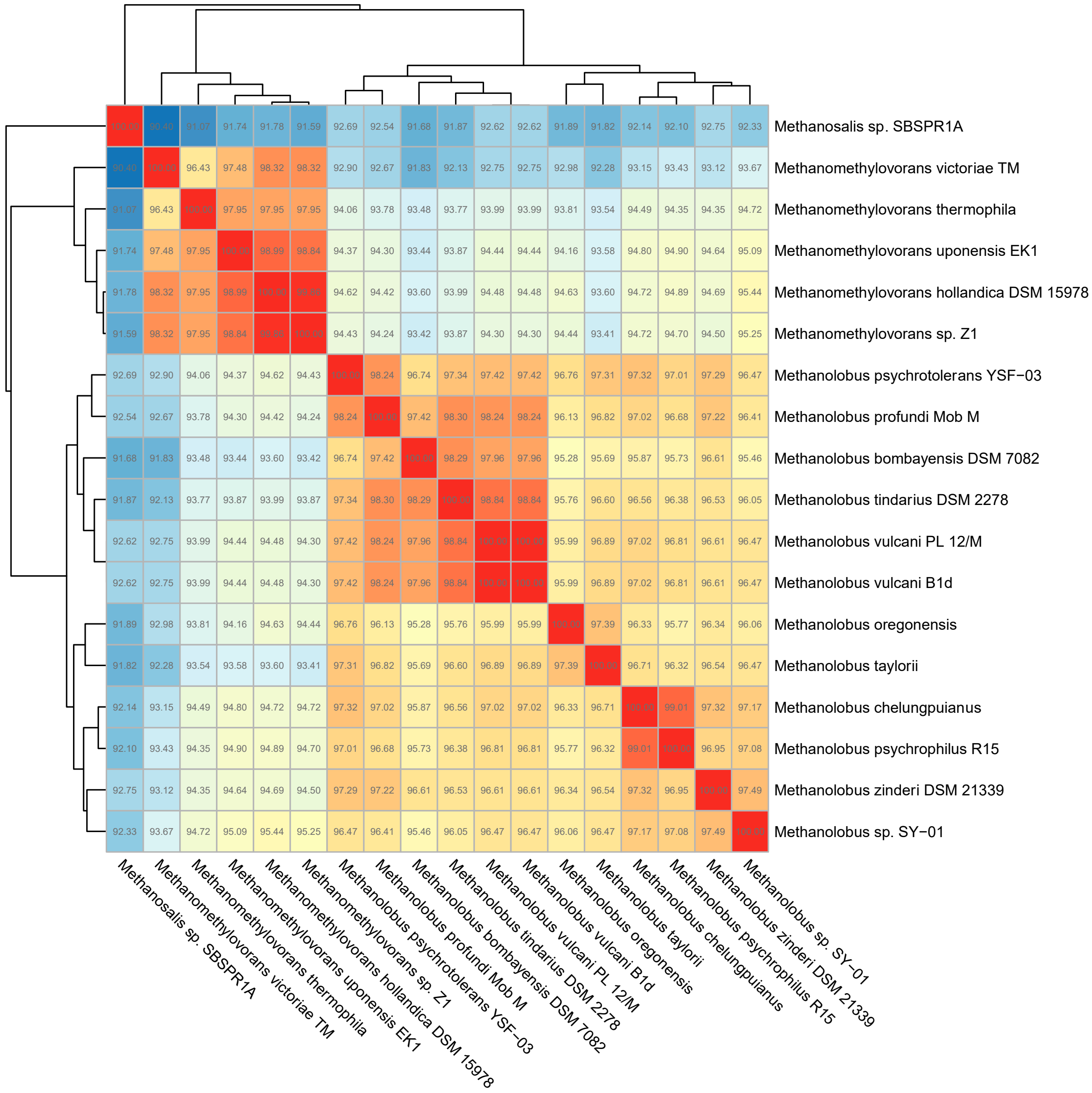

Figure S4. Heatmap of pairwise percent similarity of full length 16S rRNA genes. Dendrograms on the sides only display clustering of the percentages and do not reflect a phylogenetic analysis.

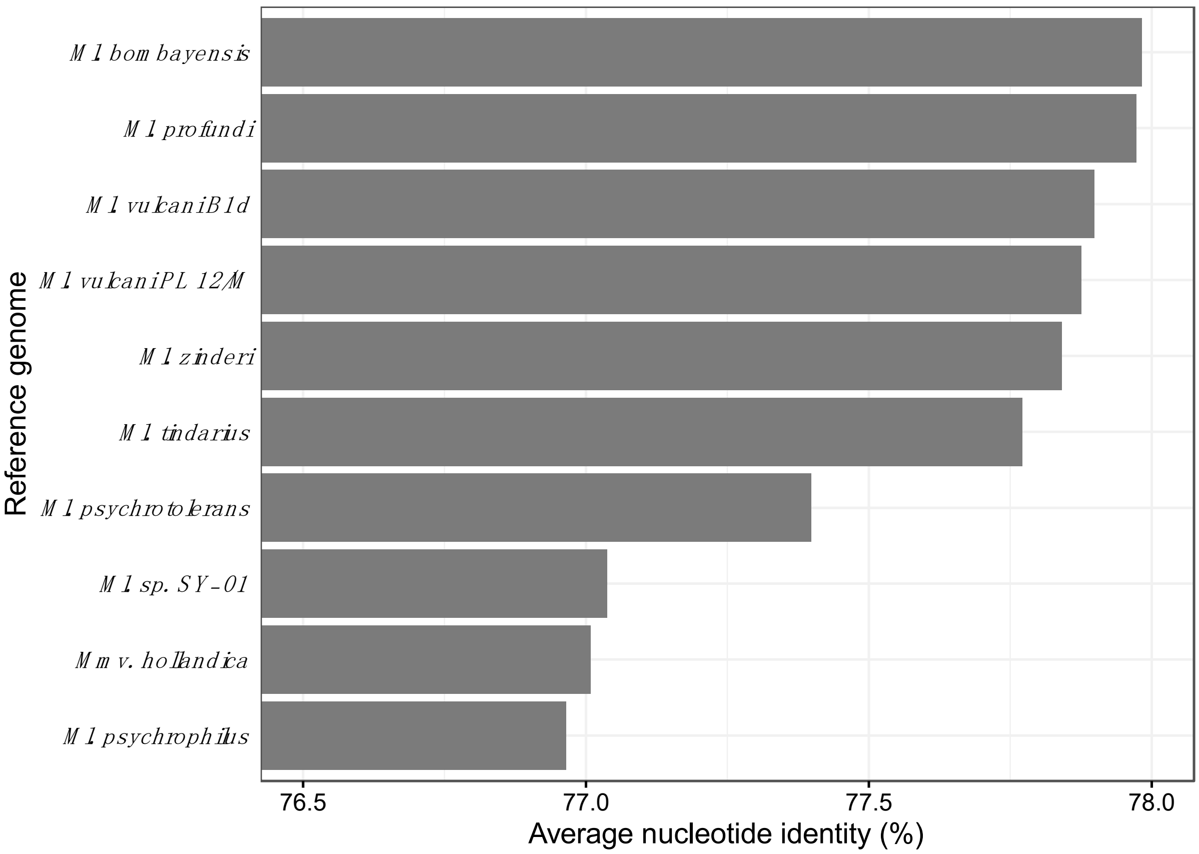

Figure S5. Average nucleotide identity (ANI) between the new Methanosalis sp. SBSPR1A genome, nine other Methanolobus reference genomes, and Methanomethylovorans hollandica. ANI was calculated with the FastANI tool in Kbase.

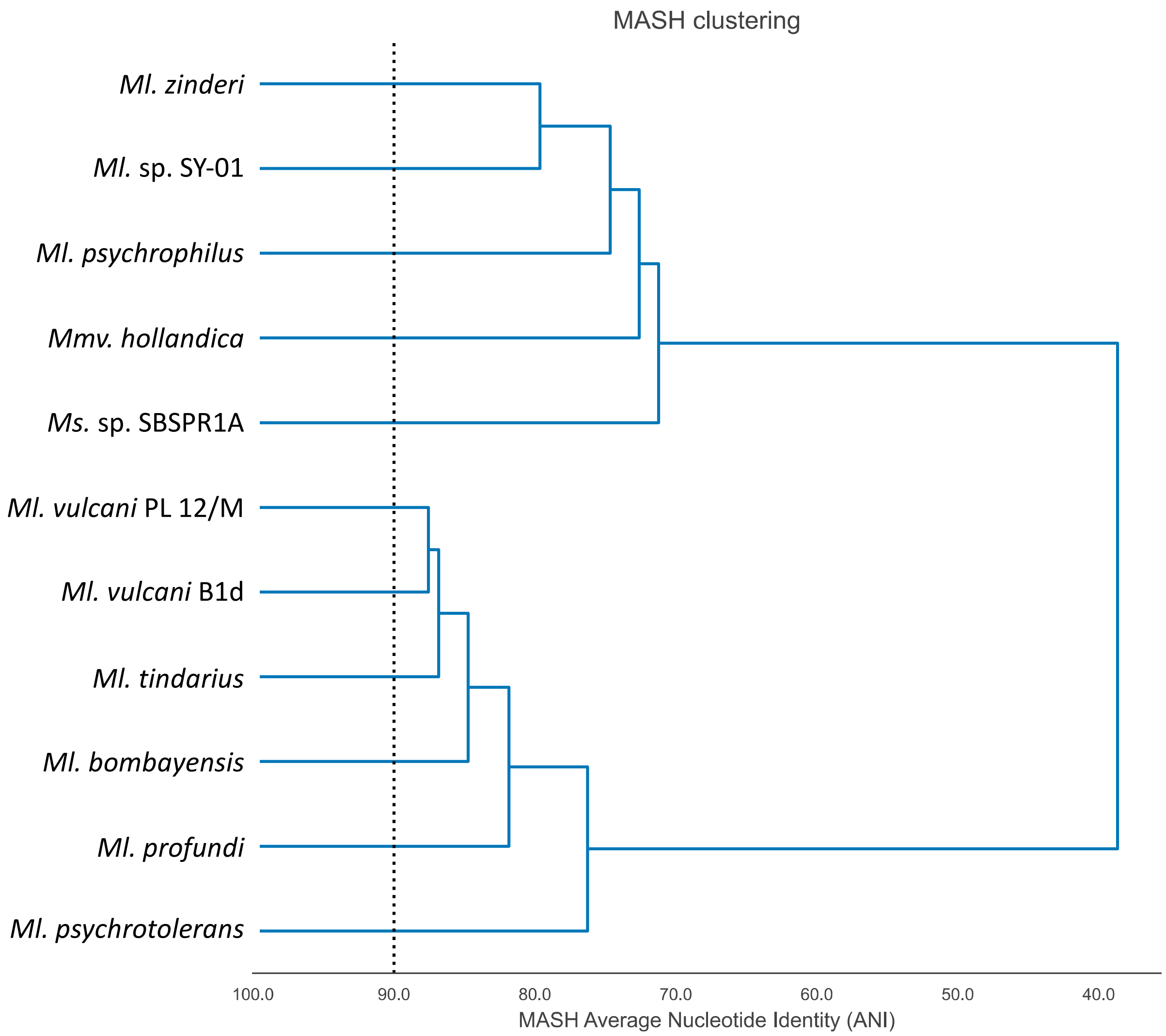

Figure S6. MASH clustering of average nucleotide identities among the 11 genomes in this study. ANIs were calculated with gANI in dRep.

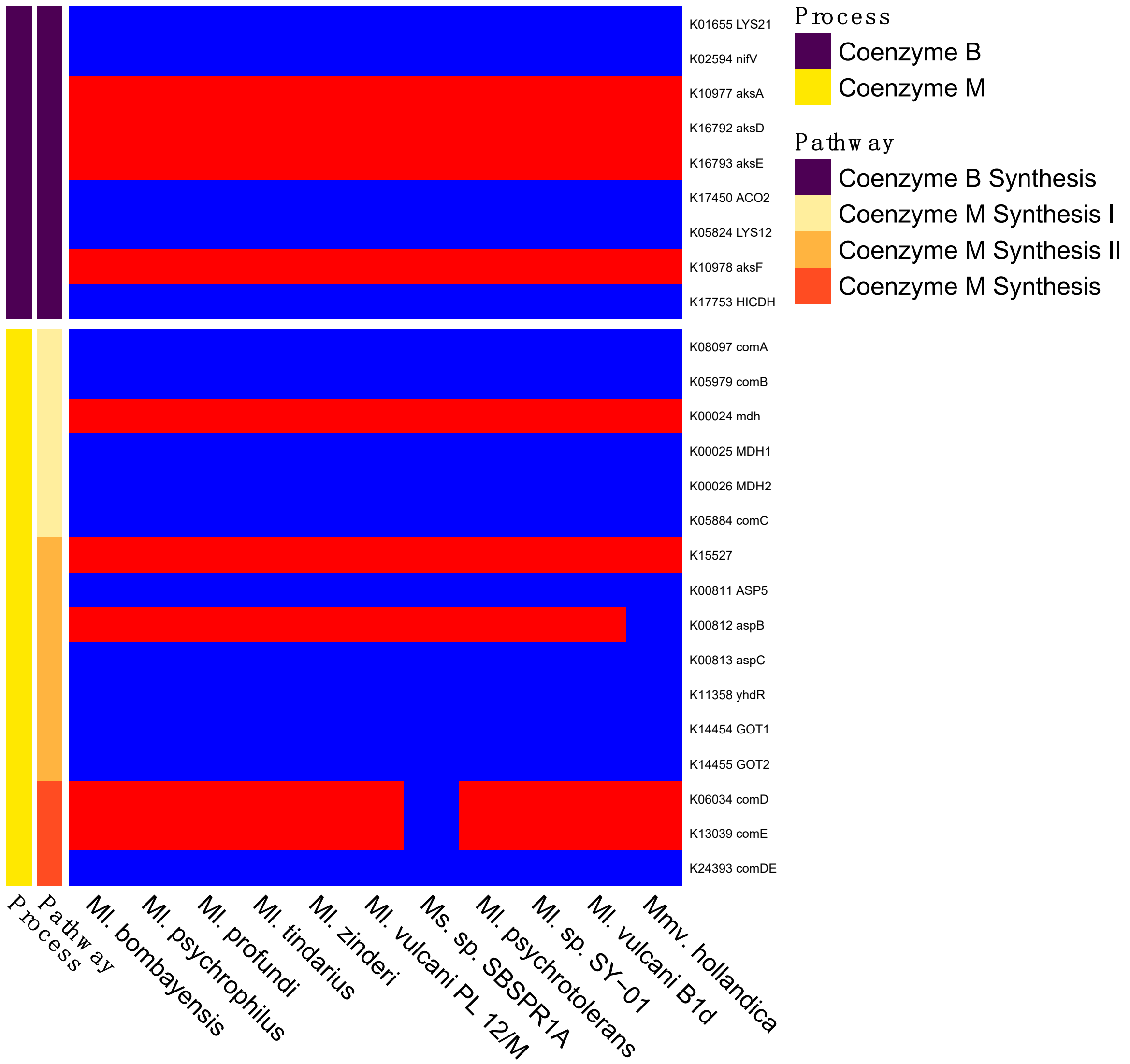

Figure S7. Presence (red) or absence (blue) of genes involved in coenzyme B and coenzyme M synthesis, two important compounds in methanogenesis. Coenzyme M synthesis can occur via two pathways, both of which produce 3-sulfopyruvate, which is then converted to coenzyme M using comD, comE, and comDE. This figure shows all of the annotated KOs involved in these pathways but note that there are several other reactions that are not shown here as they currently do not have annotated KOs.

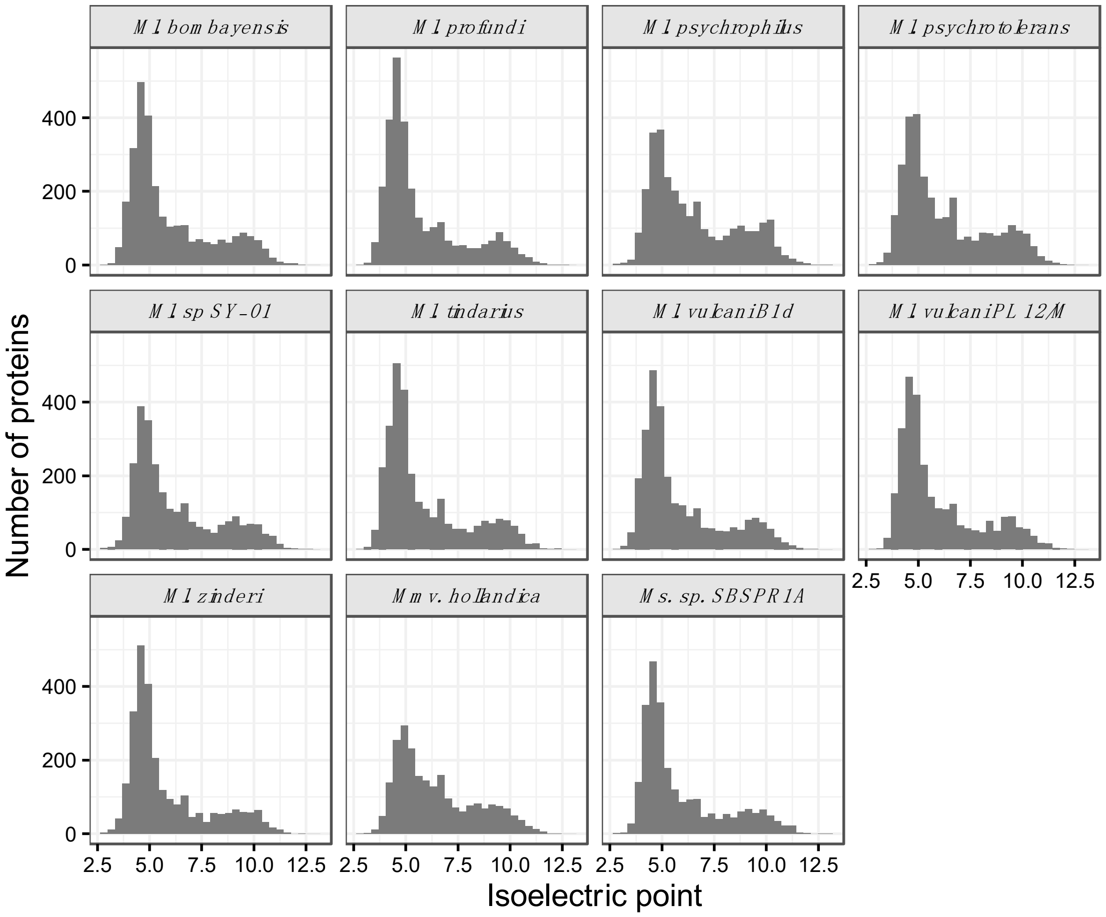

Figure S8. Isoelectric point profiles for the 11 genomes analyzed in this study. All genomes show an acidic bias with an asymmetrical bimodal distribution. Histograms were created with 30 bins.

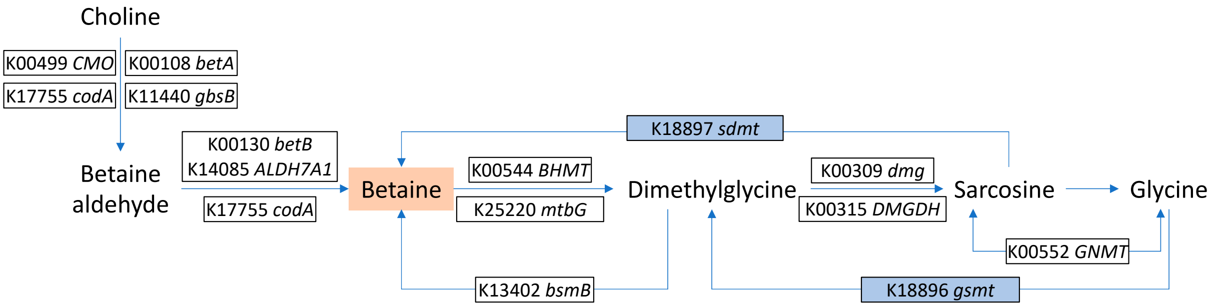

Figure S9. Betaine synthesis in Methanosalis sp. SBSPR1A, showing the presence of gsmt and sdmt genes suggesting synthesis from sarcosine, and a lack of genes for synthesis from choline. Blue cells = gene present; white cells = gene absent. Adapted from part of the KEGG reference pathway “Glycine, serine and threonine metabolism”.
